# Gender Diversity-Related Mental Health Care: Evidence, Trends, Obstacles

**DOI:** 10.1192/j.eurpsy.2024.95

**Published:** 2024-08-27

**Authors:** K. Başar

**Affiliations:** Department of Psychiatry, Hacettepe University, Ankara, Türkiye

## Abstract

**Abstract:**

Gender identity may be experienced within a broad spectrum beyond the binary understanding of sex concerning genital characteristics. In people with gender identities not congruent with the gender culturally associated with the sex assigned at birth, distress related to biopsychosocial correlates of this condition may arise. In current diagnostic systems, this is considered within the framework of “Gender Incongruence” (ICD-11) and “Gender Dysphoria” (DSM 5). Although this diversity is known to be present throughout the ages, the terms related to gender identity were introduced to medical literature a hundred years ago. They were popularized with the advances in medical procedures that assist individuals in acquiring physical features aligning with their gender identity and expression. There has been an increase in research interest with increasing numbers in medical centers working on gender-affirmative medical procedures. Starting from the 1970s, international organizations prepared guidelines on the standards of care for trans and gender diverse (TGD) individuals. Despite all the progress in the gender-affirming medical care provided to TGD individuals and the changes in the legal recognition of gender, health inequalities persist globally. The discrepancy in mental and physical health conditions has long been shown to be associated with “minority stress.” The minority stress perspective suggests that distal and proximal chronic stressors arising from society are associated with adverse health outcomes for TGD individuals. Resilience against these stressors is more robust with better coping styles and social support. Lately, structural stigma and discrimination have been shown to be an important source of inequality. Therefore, much more progress is still required with respect to societal inequalities, human rights, and structural transphobia for the improvements in medical care to impact the global health condition of TGD people. However, lately, there have been attempts to restrict TGD individuals’ access to medical care and their legal rights, even if they were not close to the level they ought to be. This backlash mostly sits on the discussion on the management of TGD adolescents and children. Models of care for these age groups have been developed for decades, and despite evidence of the protective and beneficial effects on health and development, in many countries, there are attempts to block their access to medical care. Growing debate on TGD care turned into a political combat, where scientific evidence and human rights perspectives are often ignored. These tendencies present a strong challenge for public health and the professional identity and practice of healthcare professionals.

**Disclosure of Interest:**

None Declared

